# Influence of Bond Characterization on Load-Mean Strain and Tension Stiffening Behavior of Concrete Elements Reinforced with Embedded FRP Reinforcement

**DOI:** 10.3390/ma15030799

**Published:** 2022-01-21

**Authors:** Marta Baena, Cristina Barris, Ricardo Perera, Lluís Torres

**Affiliations:** 1 Advanced Materials and Analysis for Structural Design (AMADE), Polytechnic School, University of Girona, 17003 Girona, Spain; marta.baena@udg.edu (M.B.); cristina.barris@udg.edu (C.B.); 2 Department of Mechanical Engineering, Technical University of Madrid, 28006 Madrid, Spain; ricardo.perera@upm.es

**Keywords:** materials’ characterization, bond-slip, tension stiffening, FRP composites, concrete, numerical model, parametric study, structural application

## Abstract

Based on the characterization of the bond between Fiber-Reinforced Polymer (FRP) bars and concrete, the structural behavior of cracked Glass-FRP (GFRP)-Reinforced Concrete (RC) tensile elements is studied in this paper. Simulations in which different bond-slip laws between both materials (FRP reinforcement and concrete) were used to analyze the effect of GFRP bar bond performance on the load transfer process and how it affects the load-mean strain curve, the distribution of reinforcement strain, the distribution of slip between reinforcement and concrete, and the tension stiffening effect. Additionally, a parametric study on the effect of materials (concrete grade, modulus of elasticity of the reinforcing bar, surface configuration, and reinforcement ratio) on the load-mean strain curve and the tension stiffening effect was also performed. Results from a previous experimental program, in combination with additional results obtained from Finite Element Analysis (FEA), were used to demonstrate the accuracy of the model to correctly predict the global (load-mean strain curve) and local (distribution of strains between cracks) structural behavior of the GFRP RC tensile elements.

## 1. Introduction

The use of Fiber-Reinforced Polymer (FRP) materials as an alternative to traditional steel reinforcement for Reinforced Concrete (RC) structures has been promoted in the last decades, especially in corrosive environments or when the effects of electromagnetic fields may be present. However, different FRP products are available in the market, whose properties may have a significant effect on the structural performance of the RC element. In particular, the relatively low modulus of elasticity when compared with steel, especially for Glass Fiber-Reinforced Polymers (GFRP), leads to larger crack widths, and serviceability requirements often govern the design of FRP RC elements [1].

Suitable modelling of the deformational cracking and post-cracking behavior of RC elements needs to correctly account for the tension stiffening effect. Due to the interaction between reinforcement and concrete, the intact tensioned concrete between adjacent cracks is able to sustain certain level of tensile stresses, thus contributing to the stiffness of the RC element [2,3,4]. Therefore, the deformation characteristics of cracked RC elements is dependent on the bond forces that appear between reinforcement and concrete. In this sense, bond behavior of steel bars embedded in concrete has been largely analyzed and accepted expressions are proposed in design codes [5]. Additionally, mechanical and bond properties of steel reinforcing bars have been standardized. However, this is not the case for GFRP bars, and different surface configurations (including smooth, sand coated, and ribbed surface, among others) and combinations of fibers and matrices (that yield to different modulus of elasticity) have been proposed. As a result, numerous experimental studies have been conducted to investigate the bond between FRP bars and concrete as well as the influence of bar diameter, fiber type, and surface configuration [6,7,8,9,10,11,12,13,14].

Several approaches exist to account for the tension stiffening effect as, for instance, the use of an effective cross-sectional area for tensile elements based on the combination of the uncracked and cracked cross-sectional area [15] or the attribution of additional stresses to reinforcing materials or concrete by modification of their constitutive equation [16,17,18,19,20,21,22]. Although being largely used, singularities of the interaction between the reinforcing bar and the concrete (i.e., the bond-slip law) cannot be accounted for in these models. As a more general and direct alternative, numerical models based on the finite element analysis (FEA) have been developed in recent years in which the bond-slip law may be directly included in the modelling strategy. This latter approach traditionally implies the development of a specific finite element, the use of cohesive elements, and/or the assumption of damage models, among other strategies. Associated complex simulations and large computational costs can be, however, considered as negative factors [23].

In this paper, a comparative study about the effect of different GFRP bar types (with different bond performance) on the structural behavior of GFRP RC tensile elements is presented. To this end, a numerical model for cracked elements based on bond stress-slip behavior between materials was used, in which any experimental bond-slip law can be assumed. Previous to the study, the numerical model was validated through the comparison of numerical predictions with experimental results and FEA predictions. The study of the effect of type of GFRP bar (mechanical properties and surface configuration) was thereafter followed by a parametric study on the effect of concrete grade and reinforcement ratio on the load-mean strain curve and tension stiffening effect.

## 2. Numerical Non-Linear Model for FRP-Reinforced Tensile Members

**Figure 1:**
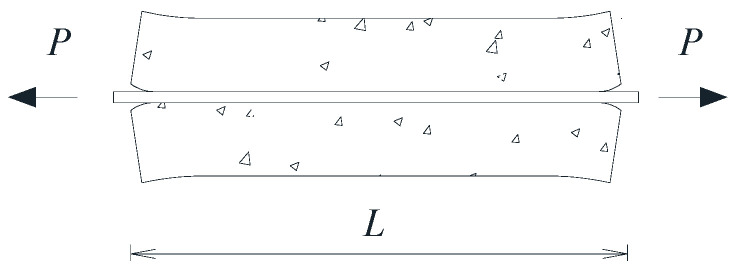
RC tensile element.

The numerical model used for the study carried out in this work on the deformational behavior of FRP-reinforced tensile members was based on the solution of the differential equation that describes the bond-slip behavior of a RC member, applied between two adjacent cracks at a distance *L*, subjected to uniaxial tensile force *P* (see Figure 1).

For the development of the numerical model, the following assumptions were considered: (1) the elastic behavior of the reinforcing bar and concrete under serviceability conditions and (2) strains, stresses, and displacements were examined along the longitudinal direction of the composite element, and these values were assumed not to vary with the radial coordinate. Along with these assumptions, strain compatibility and equilibrium of forces at any section were also imposed, which led to the well-known differential Equation (1), where *n* is the modular ratio (defined as the ratio between the modulus of elasticity of embedded reinforcement, *E_r_*, and that of concrete, *E_c_*), *ρ* is the reinforcement ratio (defined as the ratio between the cross-sectional area of reinforcement, *A_r_*, and that of concrete, *A_c_*), *p_r_* is the perimeter of the embedded reinforcement, and *τ*(*x*) and *s*(*x*) are two unknown functions, which are related through the bond-slip law.


(1) $$\frac{d^{2}s(x)}{dx^{2}}=\frac{\tau (x)p_{r}}{A_{r}E_{r}}(1+n\rho )$$

In the solution process, the definition of a bond-slip law is fundamental because it will describe the interaction between the reinforcing bar and the concrete. The capability of the numerical non-linear model to use any bond-slip law allows for its application to any type of reinforcement and, therefore, to consider the singularities of the interaction when material different to steel is used.

The finite difference method was applied to solve the problem. It should be mentioned that, within the numerical procedure, cracks were assumed to appear at the halfway section between two existing cracks. Therefore, after first cracking, two new blocks with shorter length were considered, and load was increased until new cracking was attained. More details can be found in [24]. As a result of the numerical procedure, the distribution of strains, slip, and bond stresses between both materials along the length of the member were found. Additionally, the tensile behavior of the member, in terms of load-member mean strain curve, was also obtained and tension-stiffening effect could be derived.

## 3. Numerical Model Validation

With the aim at confirming that the numerical model was suitable for the proposed study on the structural behavior of GFRP RC tensile elements, the model was validated for this section. To this end, a comparison of predictions with experimental results and results from FEA are presented.

### 3.1. Available Experimental Results

Experimental results were obtained from an experimental program on GFRP RC ties [25]. The reinforcement consisted of one GFRP bar having a helical, wrapped surface and some sand coating. Two different concrete sections (170 mm × 170 mm and 110 mm × 110 mm) and three different nominal bar diameters (13 mm, 16 mm, and 19 mm) were combined, thus giving four different reinforcement ratios (*ρ* = 0.51%, 0.71%, 1.00%, and 1.69%). The ties were casted with concrete, having a target compressive strength of 50 MPa, and were 1200 mm length. The test matrix (Table 1) included an additional specimen (16-170-3N) with three perimeter notches (equally spaced from each other), whose reinforcing bar was internally instrumented with strain gauges so that monitoring of the strain distribution along the reinforcing bar was possible [25].

### 3.2. Available FEA Results

To further check the adequacy of the performance of the numerical model, numerical predictions using Finite Element Analysis of the previously presented experimental GFRP RC tensile elements were also used. For the FEA analysis, the inverse method was used to simplify the complexity of the behavior in the interface between both materials into two main components (friction and mechanical interaction), which were thereafter modelled by using surface-to-surface contacts and connector elements, respectively. More details on the proposed methodology can be found in [26].

### 3.3. Validation

Experimental results (hereinafter referred to as Exp) and numerical predictions of the FEA analysis (hereinafter referred to as FEA) were used to demonstrate the accuracy of the more simplified numerical non-linear model (hereinafter referred to as FDM) and assumptions to correctly predict the structural behavior of the GFRP RC tensile elements. Predictions of the structural behavior were checked at two levels: (1) the local level, through the comparison of the distribution of the reinforcement strain along the length of the RC tensile element at different load levels, and (2) the global level, through the comparison of the load- mean strain curves.

**Figure 2:**
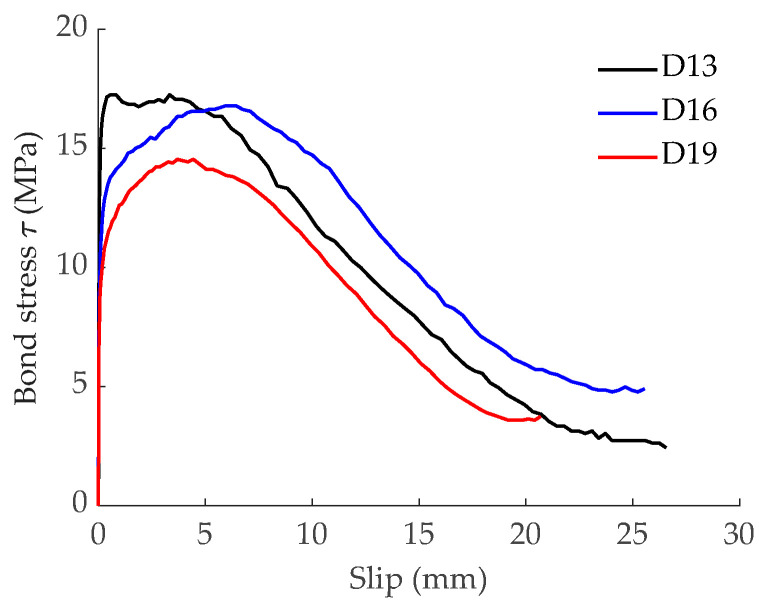
Experimental bond-slip laws.

Experimental bond-slip laws used for both the FDM model and the FEA analysis were obtained in an experimental program on pull-out tests [8]. Bond-slip curves for GFRP bars with nominal diameters of 13 mm, 16 mm, and 19 mm are shown in Figure 2. The target compressive strength of concrete was 50 MPa.

#### 3.3.1. Reinforcement Strain Distribution along the Length of the RC Tensile Element

The validation through comparison of the reinforcement strain distribution obtained from the bond-slip law was only feasible in the case of the RC tensile element with three perimeter notches, where cracks were forced to appear at specific sections (reference 16-170-3N in Table 1). With the aim at having comparable results, the experimental order and the location of cracks’ appearance as well as the load causing those cracks were imposed in the numerical simulations (FDM and FEA).

**Figure 3:**
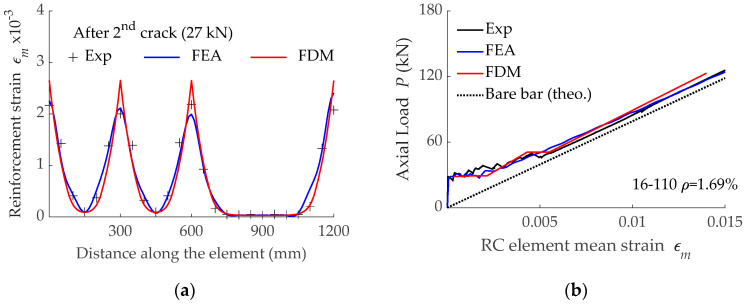
Comparison between results obtained with the non-linear model (FDM) and experimental (Exp) and numerical results using FEA: (**a**) Distribution of reinforcement strain after formation of the second crack in specimen 16-170-3N; (**b**) Load-mean strain curves for specimen 16-110.

Comparison between experimental results and numerical predictions is shown in Figure 3a. The peaks in the reinforcement strain corresponded to the existence of a crack at that section. From crack sections on, strains in reinforcement started to decrease due to the bond forces acting between reinforcement and concrete. In cases where the bonded length was larger than the length required to transfer forces from reinforcement to concrete, a plateau in the reinforcement strain appeared, thus indicating that composite action was completely recovered. This was the case, for instance, of sections allocated at 750 ≤ x ≤ 1050 mm. Results presented in Figure 3a demonstrate that the non-linear model (FDM) correctly predicted the reinforcement strain distribution. Although results are only presented for the opening of the second crack, good accuracy was obtained throughout the cracking process. The comparison in Figure 3a shows that FDM and FEA methodologies used in this work, incorporating the obtained experimental bond-slip laws, correctly reflected the actual behavior of the bonded elements.

#### 3.3.2. Load-Mean Strain Curve

For the validation of the prediction of the global structural behavior, the rest of the specimens with no perimeter notches were used (references 13-170, 16-170, 19-170, and 16-110 in Table 1), and bond-slip laws presented in Figure 2 were assumed. In this case, there was no need of imposing the cracking loads and order of cracks’ appearance. Therefore, the non-linear model (FDM) applied the assumed simplification of a crack appearing always at the halfway section between two existing cracks.

The experimental load-mean strain curve was compared with those predictions obtained with numerical simulations (FDM and FEA) in Figure 3b. Due to space limitations, results are only presented for specimen 16-110, but similar good accuracy was found in them all. An initial stage representing the elastic behavior of the RC uncracked tensile element was visible before the first cracking load was reached. The elastic behavior range was followed by the crack formation stage where primary cracks appeared. This is why this stage is also known as the primary cracking stage. It should be noted that the appearance of new cracks was represented in the FDM simulations by a sudden increase in the mean strain at a constant force value. Finally, the crack formation stage was followed by the stabilized cracking stage, where no more cracks appeared and the response of the RC tensile element approached that of the bare reinforcement. Similar to the prediction of the local behavior, results (in Figure 3b) confirmed the capability of the non-linear model (FDM) to correctly predict the load-mean strain curves.

Therefore, the non-linear model (FDM) was demonstrated to be an effective tool for the analysis of the cracking process of RC tensile elements, both at the local and global levels, allowing us to include the effect of bond-slip constitutive laws. Additionally, the simpler FDM model had a reduced computational cost when compared to FEA. Besides, the tension stiffening effect (the ability of tensioned concrete between cracks to sustain a certain level of stresses) can be derived from the load-mean strain curve. This way, the FDM non-linear model was used for the next sections for studying the possible effect of the variables involved in the deformational behavior of the FRP RC tensile elements.

## 4. Effect of Different Types of GFRP Bars on the Performance of Cracked RC Elements

Different types of GFRP bars exist in the market. They may differ in their mechanical properties (tensile strength and modulus of elasticity) and on the surface configuration used to provide the bond interaction with the surrounding concrete. In fact, different bond mechanisms are activated depending on the surface configuration of the reinforcing bar and on the confinement provided by concrete.

As a result of the lack of standardization of the surface characteristics, several studies have been conducted in which bond behavior between GFRP bars and concrete was analyzed and different bond-slip law were reported [6,7,8,9,10,11,12,13,14].

In this section, the effect of the bond performance of the GFRP bar on the structural response of GFRP RC tensile elements was analyzed. To this end, the FDM model was used to simulate three GFRP RC tensile elements, which differed in the type of GFRP reinforcing bar assumed. The modelled GFRP-reinforced tensile elements were 1200 mm length and had a 110 mm × 110 mm concrete section with one centered GFRP bar having a nominal diameter of 16 mm, thus giving a reinforcement ratio equal to *ρ* = 1.69%.

**Figure 4:**
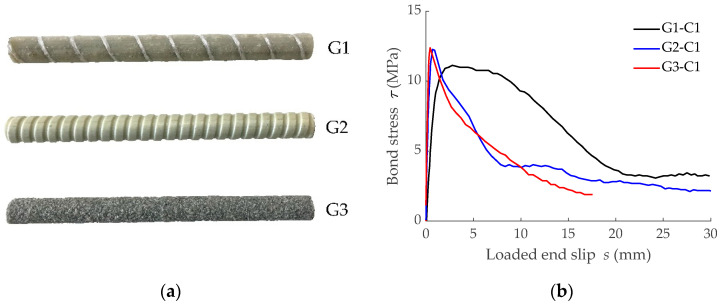
Information on the GFRP bars: (**a**) Detail of GFRP bars’ surface configuration; (**b**) Bond-slip laws between the GFRP bars and low concrete grade (C1) [8].

Three different GFRP bars with different surface characteristics were considered: (1) GFRP bar with a helical wrapping surface and some sand coating, referred to as G1; (2) GFRP bar with a grooved surface, referred to as G2; and (3) GFRP bar with a sand-coated surface, referred to as G3 (see Figure 4a). Experimental bond-slip laws corresponding to bars G1, G2, and G3 embedded in low concrete grade (C1, with target compressive strength of 25 MPa) were obtained in [8] and are presented in Figure 4b. For the numerical simulations, a mean concrete tensile strength, *f_ctm_*, of 2.2 MPa and a modulus of elasticity, *E_cm_*, of 30 GPa were assumed, which corresponded to an experimental mean compressive strength, *f_cm_*, equal to 28 MPa, according to Eurocode 2 [5]. Finally, values for the modulus of elasticity of the reinforcing bar given by the manufacturers were used (i.e., *E_r_* equals 40.8 GPa, 60 GPa, and 46 GPa for G1, G2, and G3, respectively).

### 4.1. Effect on Load-Mean Strain Curve

**Figure 5:**
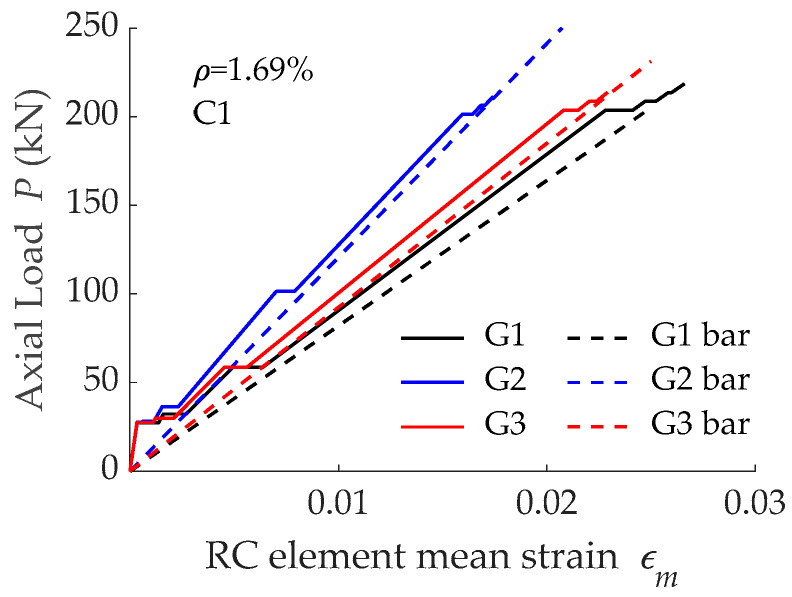
Load-mean strain curves obtained with different GFRP reinforcing bars (having different surface configurations) embedded in concrete C1.

The predicted load-mean strain curves along with the corresponding bare bar curves are presented in Figure 5, where the three stages of the cracking process can be distinguished (initial elastic, crack formation stage, and stabilized cracking stage). From numerical predictions it can be observed that loads causing formation of the successive cracks (Table 2) were different for each element and the shift from the crack formation stage to the stabilized cracking stage took place at different loads, as a consequence of the evolution of the bond forces’ transmission process between reinforcement and concrete. Numerical results on the specimen reinforced with bar G1 showed that only three cracking processes could take place during this crack formation stage, while specimens reinforced with G2 and G3 bars could undergo four cracking processes. This is a sign of the lower capacity of the bonded joint (in G1) to transfer forces between concrete and reinforcement, which is in accordance with the bond-slip laws reported in Figure 4b.

### 4.2. Effect on Reinforcement Strain Distribution along the Tensile Element

**Figure 6:**
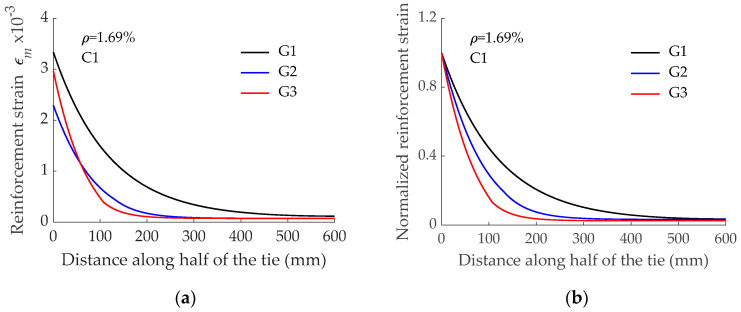
Effect of GFRP reinforcing bar surface configuration on: (**a**) Reinforcement strain distribution; (**b**) Normalized reinforcement strain distribution.

To further analyze the effect of the type of bar on the force transfer process, the reinforcement strain profile along half of the GFRP RC tensile element length is presented in Figure 6a. Data presented in Figure 6a correspond to reinforcement strains at the first cracking load level just before the formation of the crack. It should be mentioned that x = 0 mm corresponds to the end of the element and that symmetry at x = 600 mm should be applied to this data to have the distribution along the whole length. Although the three RC elements had a similar first cracking load (27.35 kN, 27.63 kN, and 27.43 kN for elements reinforced with bars G1, G2, and G3, respectively), different values for the reinforcement strain at the end of the element (acting as a cracked section) were obtained because of the different modulus of elasticity of the GFRP reinforcing bar (40.8 GPa, 60 GPa, and 46 GPa for bars G1, G2, and G3, respectively). From this section on, the reinforcement strain decreased due to the bond action. It was seen that a longer length was needed to transfer forces from reinforcement to concrete in the case of the element reinforced with bar G1. This was attributed to a less stiff initial branch of the corresponding bond-slip law (plot G1-C1 in Figure 4b). Besides, although bond-strength values for bars G2 and G3 were similar, initial stiffness in the bond-slip law corresponding to bar G3 (plot G3-C1 in Figure 4b) was relatively higher. Accordingly, a shorter length was required for the force transfer process. These tendencies are also visible in Figure 6b, where values for reinforcement strain are normalized with respect to the corresponding value at x = 0 mm.

### 4.3. Effect on Slip Distribution along the Tensile Element

**Figure 7:**
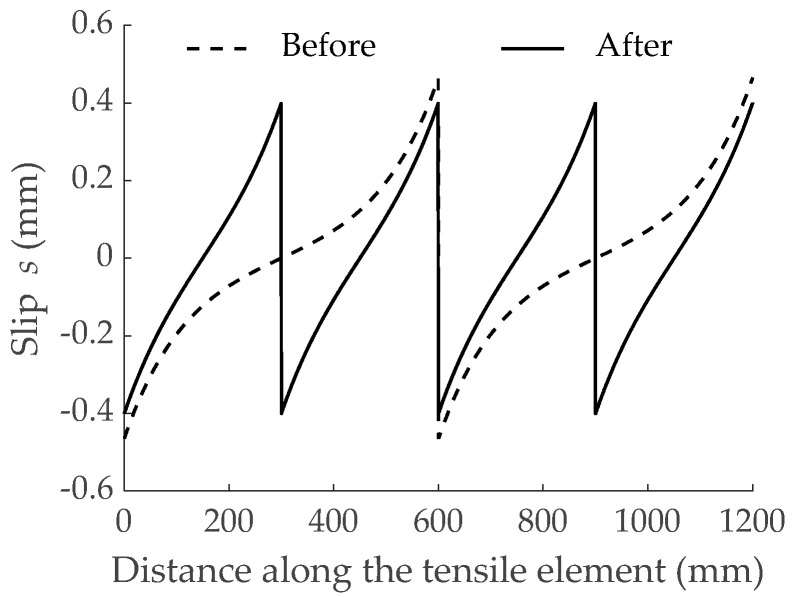
Distribution of slip before and after the second crack formation for the specimen reinforced with bar G1.

**Figure 8:**
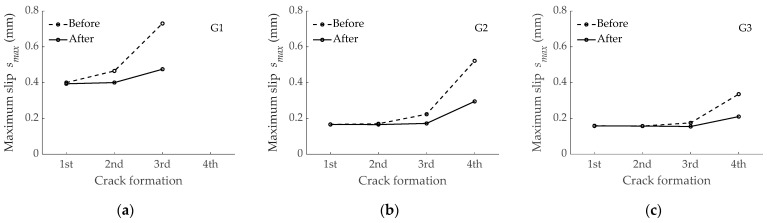
Maximum slips at the cracked section before and after the crack formation for the tensile element reinforced with: (**a**) G1 bar; (**b**) G2 bar; (**c**) G3 bar.

As an additional check of the effect of the bond performance of the GFRP bar on the force transmission process, slips obtained with the numerical simulations were also analyzed. As an example, the evolution of slips along the length of the element reinforced with bar G1 before and after the second crack formation is shown in Figure 7. The stage corresponding to the second crack formation represented the crack of the two existing blocks in two parts, thus leading to a scenario of moving from two to four reinforced concrete blocks. Finally, numerical results depicted that maximum slips appeared always at the cracked sections, as expected. Values for maximum slips, *s_max_*, before and after each crack formation are presented in Figure 8. With the aim at more easily comparing numerical results, the same scale was used in the three subfigures. The predicted maximum slips for the RC tensile element reinforced with bar G1 were larger (almost double) than those of elements reinforced with bars G2 and G3. Finally, the specimen reinforced with bar G3 showed the lowest values of slips at the cracked sections. This was again a consequence of bar G3 having the stiffer bond-slip response.

It should be mentioned that slips occurring during the crack formation stage did not exceed the slip corresponding to the bond strength (i.e., the slip corresponding to the maximum value for bond stress in the bond-slip curve). This is an indication of descending branches of the bond-slip law not being attained under service conditions.

### 4.4. Effect on Tension Stiffening Effect

**Figure 9:**
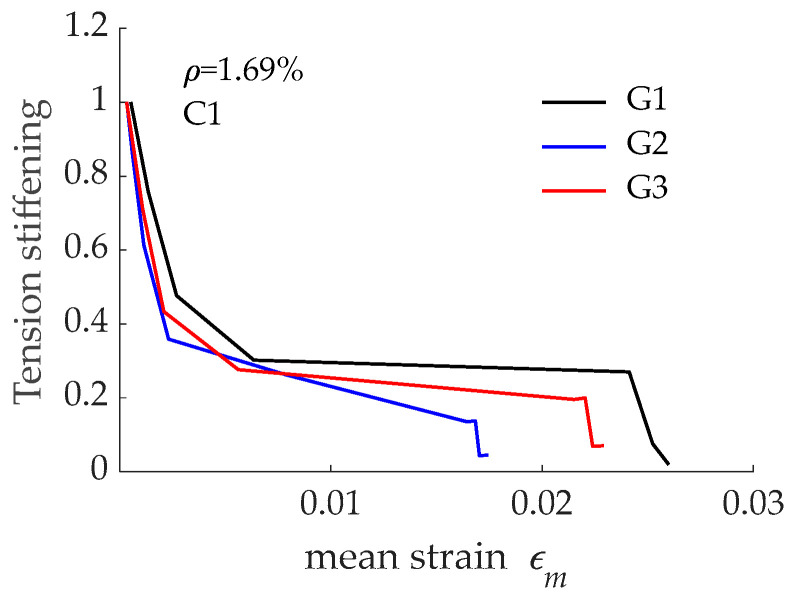
Effect of GFRP reinforcing bar bond performance on the tension stiffening effect in concrete C1.

As mentioned in previous paragraphs, the tension stiffening effect was deduced from the load-mean strain curve. The tension stiffening effect derived from curves in Figure 5 are presented in Figure 9 as the ratio between tensile stress in concrete and tensile stress in concrete at the first cracking load. Although the bond-slip curve was found to affect the load transfer process, no significant differences between the three tension stiffening curves were visible. It is acknowledged in the literature that the larger the *nρ* value, the lower the tension stiffening effect. The Reinforcement ratio (*ρ*) was constant in the presented predictions. However, this was not the case for *n*, as the three GFRP bars considered had different modulus of elasticity (40.8 GPa, 60 GPa, and 46 GPa for G1, G2, and G3, respectively, according to values available in product datasheets). This explains the lowest and larger amounts of tension stiffening for G2 and G1, respectively.

## 5. Parametric Study on Tension Stiffening Effect

### 5.1. Effect of Concrete Strength on Tension Stiffening

**Figure 10:**
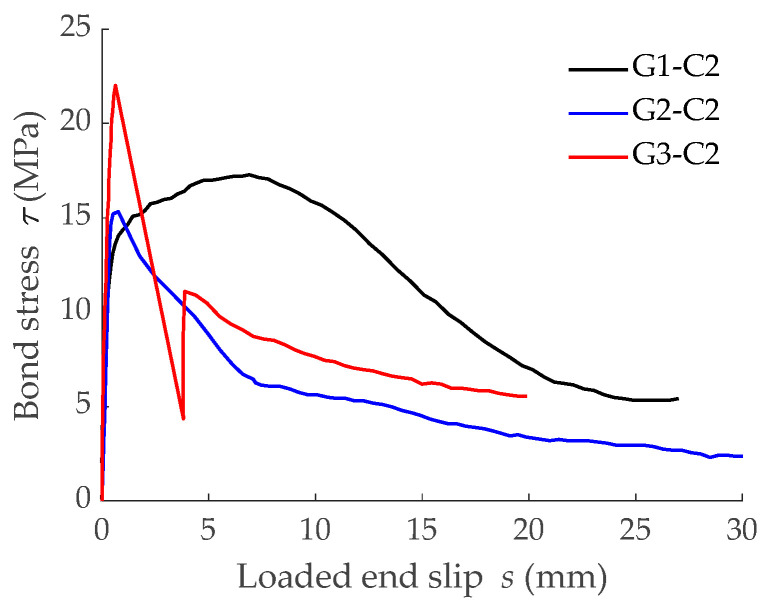
Bond-slip laws between the GFRP bars and high concrete grade (C2) [8].

In this section, the FDM model was used to analyze the possible effect of concrete strength on tension stiffening. The analysis was performed through the comparison of six numerical predictions, three of which corresponded to predictions of the previous section (with concrete C1). The three new predictions corresponded to the same geometrical characteristics but assuming a higher concrete grade (C2, with target compressive strength of 50 MPa). For the numerical simulations, an experimental mean compressive strength, *f_cm_*, equal to 54 MPa was assumed, which corresponded to a mean concrete tensile strength, *f_ctm_*, of 3.8 MPa and a modulus of elasticity, *E_cm_*, of 36.5 GPa, according to Eurocode 2 [5]. Experimental bond-slip laws implemented in the simulations for low concrete grade C1 are shown in Figure 4b, while those implemented in the simulations for higher concrete grade C2 are presented in Figure 10.

**Figure 11:**
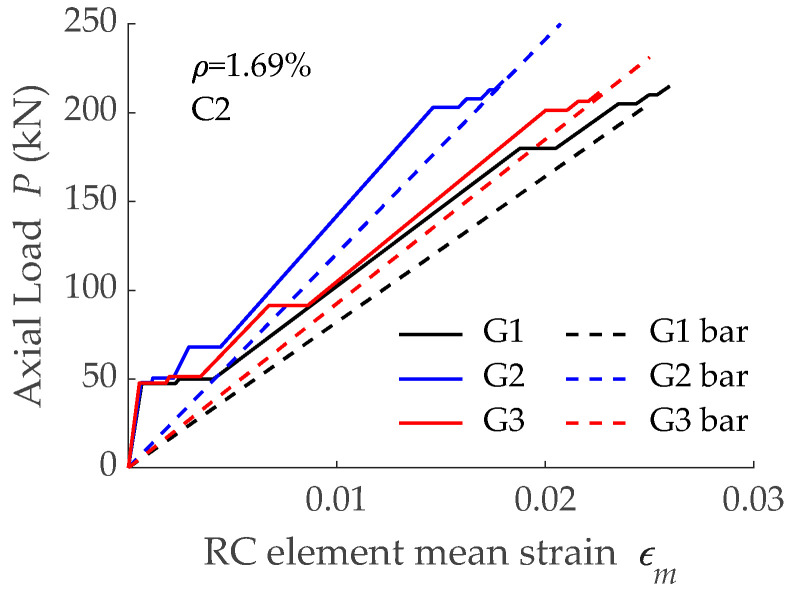
Load-mean strain curves obtained with different GFRP reinforcing bars (having different bond performance) embedded in concrete C2.

Numerical predictions on load-mean strain curves for simulations with low concrete grade (C1) are shown in Figure 5, while those for high concrete grade (C2) are presented in Figure 11. Similar to what happened for concrete C1, the three different stages of the cracking process of a RC tensile element can be distinguished in Figure 11. Focusing on the crack formation stage of C2 elements, different values of a load causing cracking (presented in Table 3) were observed. In addition, the shift from crack formation to crack stabilization stages took place for different levels of load. These results were in agreement with the numerical results of C1 elements (Figure 5 and Table 2) and were caused by the differences in the bond-slip law of bars with different bond performance (see Figure 10).

**Figure 12:**
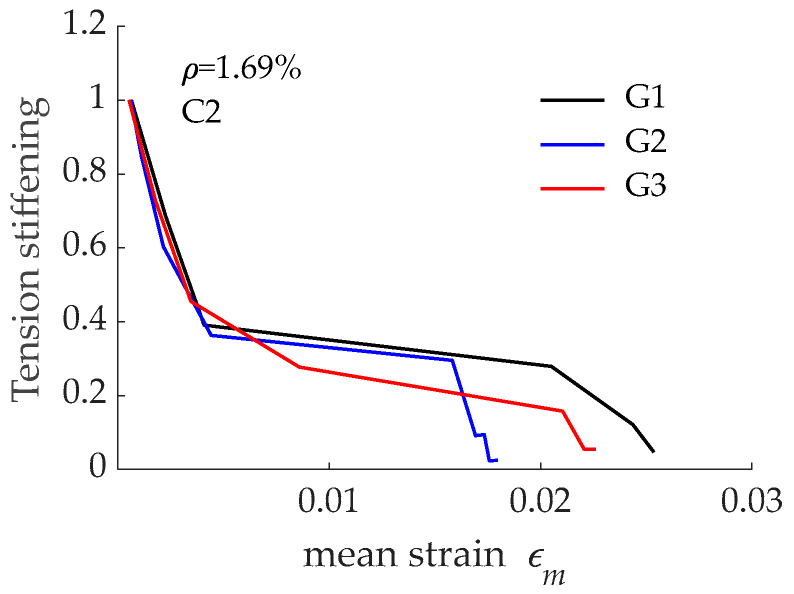
Tension stiffening effect corresponding to RC tensile elements reinforced with bars G1, G2, and G3 and concrete C2.

The tension stiffening effect derived from load-mean strain curves presented in Figure 11 is presented in Figure 12. According to numerical predictions, no significant differences existed between the three curves. The comparison between results in Figure 9 and those in Figure 12 revealed that the differences in tension stiffening even reduced when concrete compressive strength increased from C1 to C2 (i.e., from 28 MPa to 54 MPa). This was attributed to the improvement in bond performance when GFRP was embedded in concrete with higher compressive strength and to the reduction in the *nρ* value due to a larger modulus of elasticity for concrete C2 when compared to concrete C1. It can be also stated that bond performance of the reinforcing bars had no significant effect on the tension stiffening effect.

**Figure 13:**
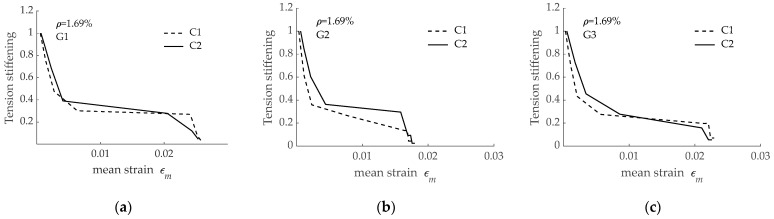
Effect of concrete compressive strength on tension stiffening effect for RC tensile elements (*ρ* = 1.69%) reinforced with: (**a**) G1 bar; (**b**) G2 bar; (**c**) G3 bar.

To better compare the effect of concrete strength on tension stiffening, and with the aim at removing any possible effect of GFRP bar surface configuration and modulus of elasticity, numerical predictions for the three different reinforcing bars are separately presented in Figure 13. According to these predictions, a larger tension stiffening effect was obtained for a larger concrete compressive strength, irrespective of the GFRP reinforcing bar used.

### 5.2. Effect of Reinforcement Ratio on Tension Stiffening

In this section, the non-linear FDM model was used to analyze the effect of the reinforcement ratio on the tension stiffening effect. Experimental bond-slip laws, presented in Figure 4b and Figure 10, were used for the numerical simulations and, consequently, the reinforcing area was a fixed parameter and the area of concrete was accordingly modified.

Predictions used for the analysis in Section 5.1 were supplemented by six more predictions, with a new reinforcement ratio *ρ*_2_ = 0.71%. Dimensions for the concrete section corresponding to *ρ*_2_ were 170 mm × 170 mm. Besides, mechanical properties for concrete were assumed to be equal to those of Section 5.1 and bond-slip laws were those presented in Figure 4b and Figure 10.

**Figure 14:**
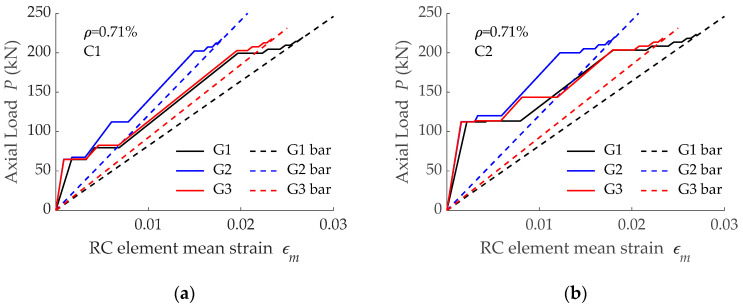
Load-mean strain curves predicted with bars G1, G2, and G3 embedded in: (**a**) concrete C1; (**b**) concrete C2.

Predictions on load-mean strain curves corresponding to specimens with a reinforcement ratio equal to 0.71% are presented in Figure 14, while those corresponding to *ρ* =1.69% are presented in Figure 5 and Figure 11 for concrete grades C1 and C2, respectively. In all cases, differences in the loads causing cracking in the crack formation stage were visible, with this being a consequence of the different load transfer process derived from different bond-slip laws. A deep analysis on this load transfer process is presented in Section 4.

**Figure 15:**
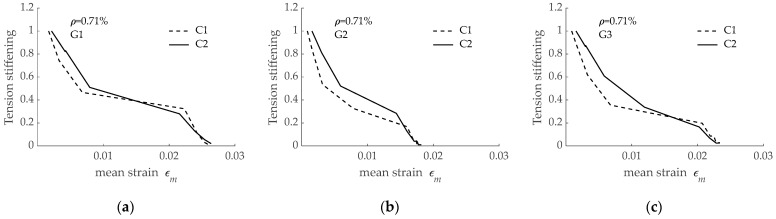
Effect of concrete compressive strength on tension stiffening effect for RC tensile elements (*ρ* = 0.71%) reinforced with: (**a**) G1 bar; (**b**) G2 bar; (**c**) G3 bar.

Based on the load-mean strain curves presented in Figure 14, the tension stiffening effect for *ρ* = 0.71% was derived and is presented in Figure 15, with the data grouped according to the reinforcing bar. It is seen that a larger tension stiffening effect was obtained for larger concrete compressive strength, irrespective of the bond-slip curve assumed. These results are in accordance with predictions with *ρ* = 1.69% presented in Figure 13.

**Figure 16:**
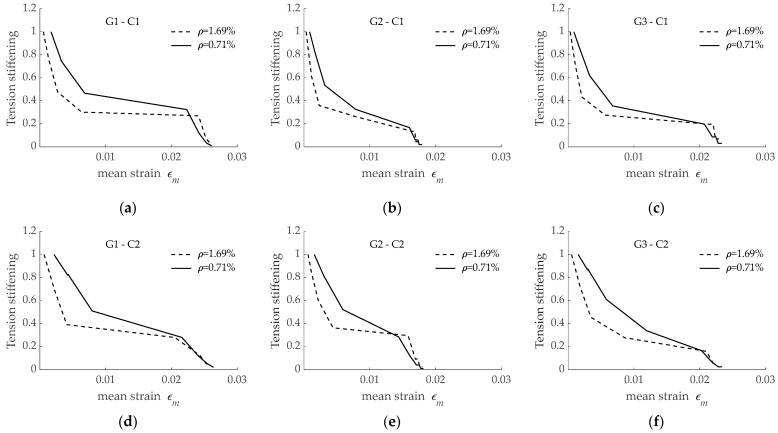
Effect of reinforcement ratio on tension stiffening predicted for: (**a**) bar G1 embedded in C1; (**b**) bar G2 embedded in C1; (**c**) bar G3 embedded in C1; (**d**) bar G1 embedded in C2; (**e**) bar G2 embedded in C2; (**f**) bar G3 embedded in C2.

Finally, numerical predictions were analyzed to determine the effect of the reinforcement ratio on the tension stiffening effect. With the aim at having representative plots, separate subfigures are presented in Figure 16 for every combination of GFRP reinforcing bar and concrete grade. According to presented results, a larger amount of tension stiffening was obtained for a lower reinforcement ratio, irrespective of the reinforcing bar and concrete grade. It should be noted that differences in the tension stiffening appeared mostly in the crack formation stage, and similar values of tension stiffening were obtained when the crack stabilization stage was reached.

## 6. Conclusions

The effect of the bond performance between different types of GFRP reinforcement (bar surface configuration consisting of helically wrapped surface with some sand coating, grooved surface, and sand-coated surface) and concrete on the deformational behavior of cracked GFRP RC tensile elements was studied in this paper. Numerical predictions of a bond-based model were used to analyze the bond transfer process and its effect on load-mean strain curve, reinforcement strain distribution, slip distribution, and tension stiffening effect. The goodness of the model to properly predict the local and global structural behavior was checked through comparison of numerical predictions with experimental results and predictions obtained with FEA. Additionally, a parametric study on the effect of concrete grade and reinforcement ratio on tension stiffening effect was performed. The following conclusions were drawn:

- Bond-slip curve of a GFRP reinforcing bar has an effect on the force transfer process taking place in the cracked RC elements (i.e., reinforcement strain and slip distributions along the length of the element) and, as a consequence, on the loads causing cracking in the crack formation stage.
- The lower bond strength and the initial stiffness in the bond-slip law (characteristic of bar G1) corresponded to a larger transfer length, required to fully recover the composite action between reinforcement and concrete, and a larger prediction of slips. The stiffer bond-slip law of bar G3 derived from a more efficient force transfer process (with shorter transfer length and slips).
- Under service conditions, the slips occurring along the RC tensile element were comparable to slips registered in the ascending branch of the bond-slip law, irrespective of the reinforcing bar.
- No significant effect of GFRP bar bond performance on tension stiffening effect was visible in the simulations with high concrete grade (C2), and differences appearing in the simulations with low concrete grade (C1) were attributed to changes in the modular ratio.
- Tension stiffening effect is proportional to concrete grade (irrespective of the GFRP embedded bar surface configuration and reinforcement ratio) and inversely proportional to reinforcement ratio (irrespective of the GFRP embedded bar surface configuration and concrete strength).
- The numerical model was validated at a local level (reinforcement strain distribution along the length of the RC tensile element) and a global level (RC tensile element load-mean strain curve), and good accuracy in its predictions was demonstrated.

## Figures and Tables

**Table 1:** Test matrix on tensile elements [25].

Specimen	Experimental Bar Diameter, *d* (mm)	Concrete Section (mm × mm)	Reinforcement Ratio, *ρ* (%)
13-170	13.7	170 × 170	0.51
16-170	16.9	170 × 170	0.71
19-170	19.1	170 × 170	1.00
16-110	16.1	110 × 110	1.69
16-170-3N	19.1	170 × 170	1.00

**Table 2:** Loads causing cracking in the crack formation stage (concrete C1).

Reinforcing Bar	Loads Causing Cracking ^1^ (kN)			
	1st	2nd	3rd	4th
G1	27.35	27.35	58.60	-
G2	27.63	28.26	36.38	101.38
G3	27.43	27.42	29.92	58.67

^1^ only cracks appearing during the crack formation stage are listed.

**Table 3:** Loads causing cracking in the crack formation stage (concrete C2).

Reinforcing Bar	Loads Causing Cracking ^1^ (kN)			
	1st	2nd	3rd	4th
G1	47.47	47.47	49.97	-
G2	47.87	50.38	67.88	-
G3	47.58	47.58	51.33	91.33

^1^ only cracks appearing during the crack formation stage are listed.
